# Supplementary figures and images for: Deciphering the preeclampsia-specific immune microenvironment and the role of pro-inflammatory macrophages at the maternal–fetal interface
Source: eLife. 2025 Mar 28;13:RP100002. doi: 10.7554/eLife.100002 (PMC11952753; doi:10.7554/eLife.100002)

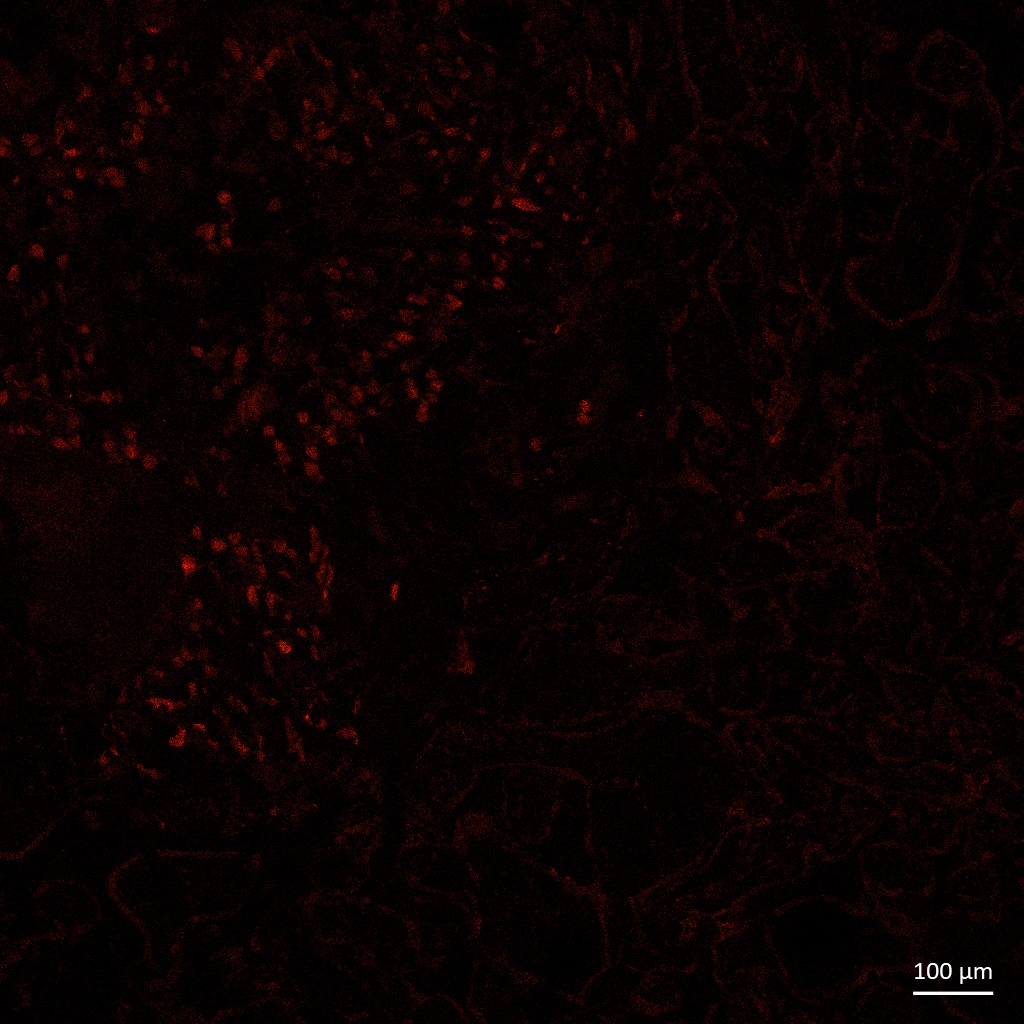

Supplement: Figure 2—source data 1. [file elife-100002-fig2-data1.zip › Figure 2-source data/figure 2D NP/NP-1.jpg]

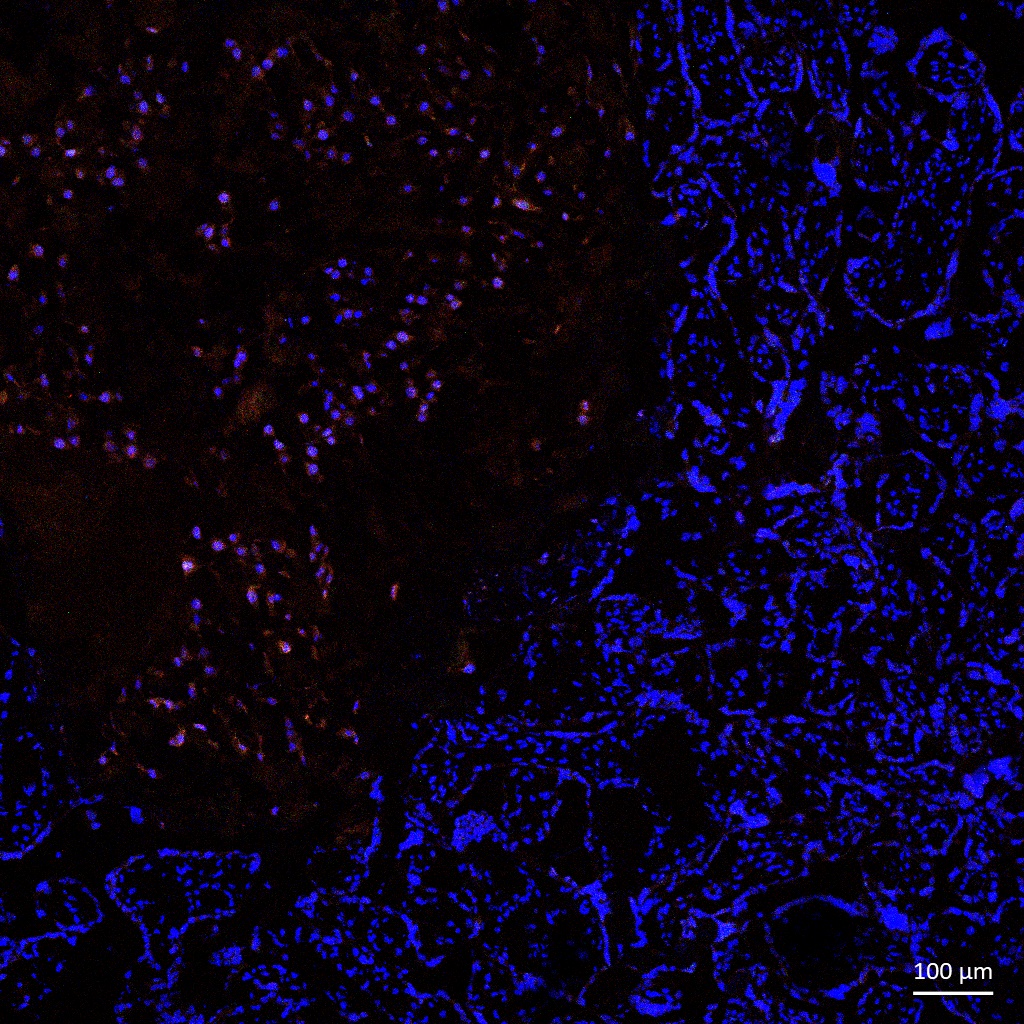

Supplement: Figure 2—source data 1. [file elife-100002-fig2-data1.zip › Figure 2-source data/figure 2D NP/NP-2.jpg]

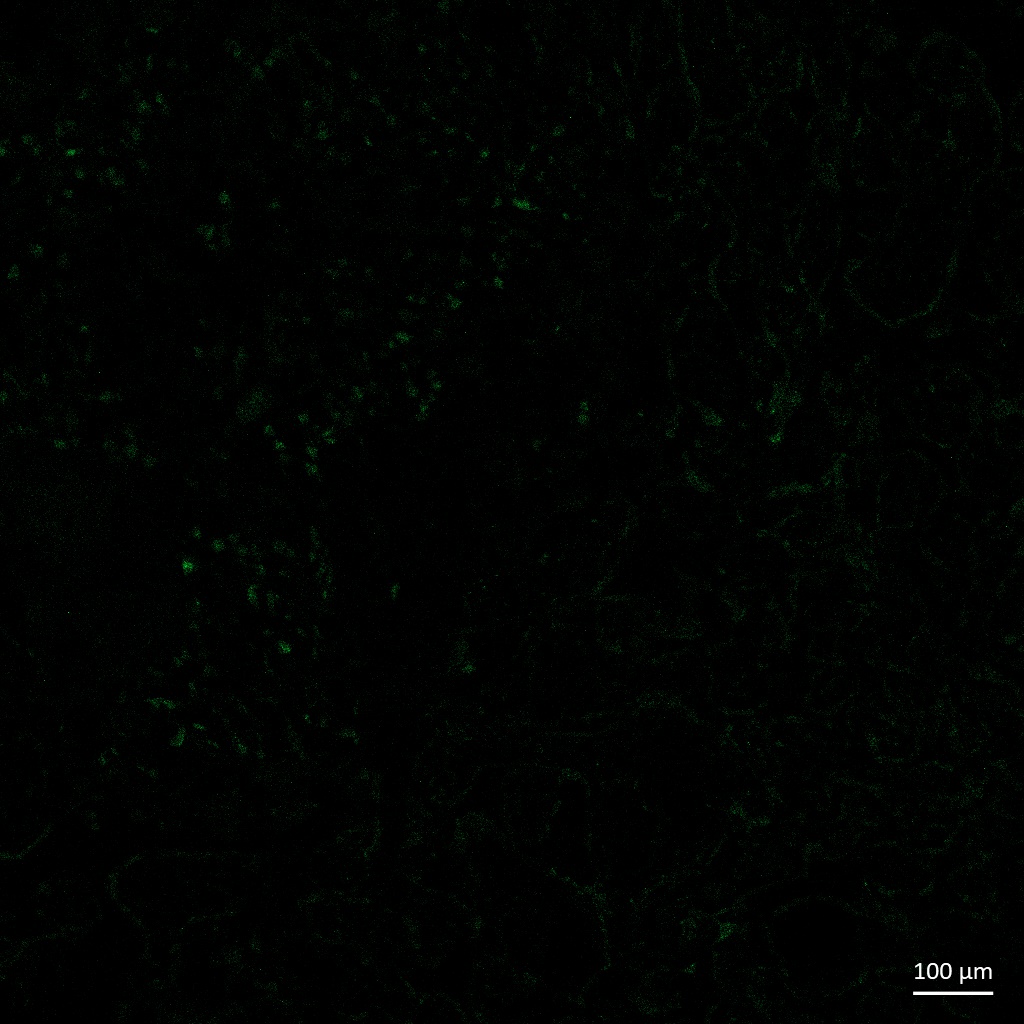

Supplement: Figure 2—source data 1. [file elife-100002-fig2-data1.zip › Figure 2-source data/figure 2D NP/NP-3.jpg]

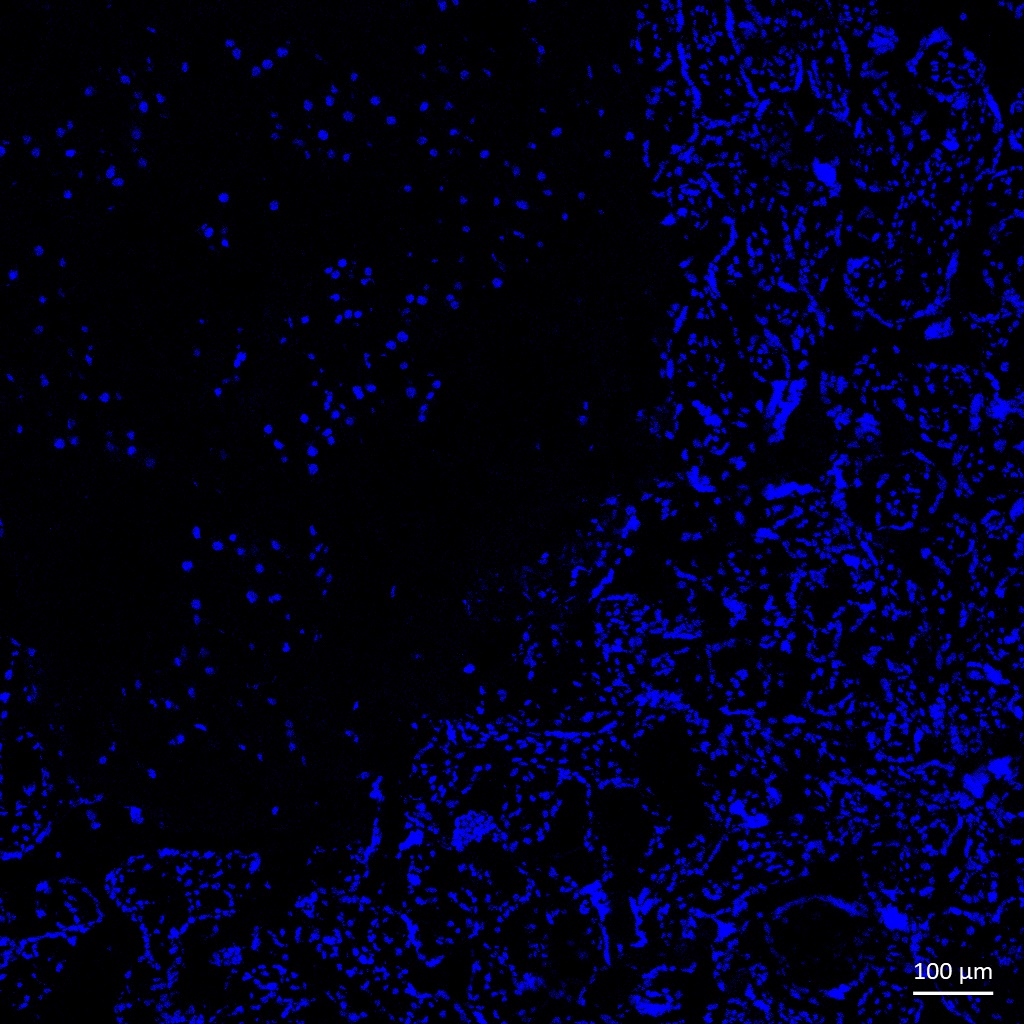

Supplement: Figure 2—source data 1. [file elife-100002-fig2-data1.zip › Figure 2-source data/figure 2D NP/NP-4.jpg]

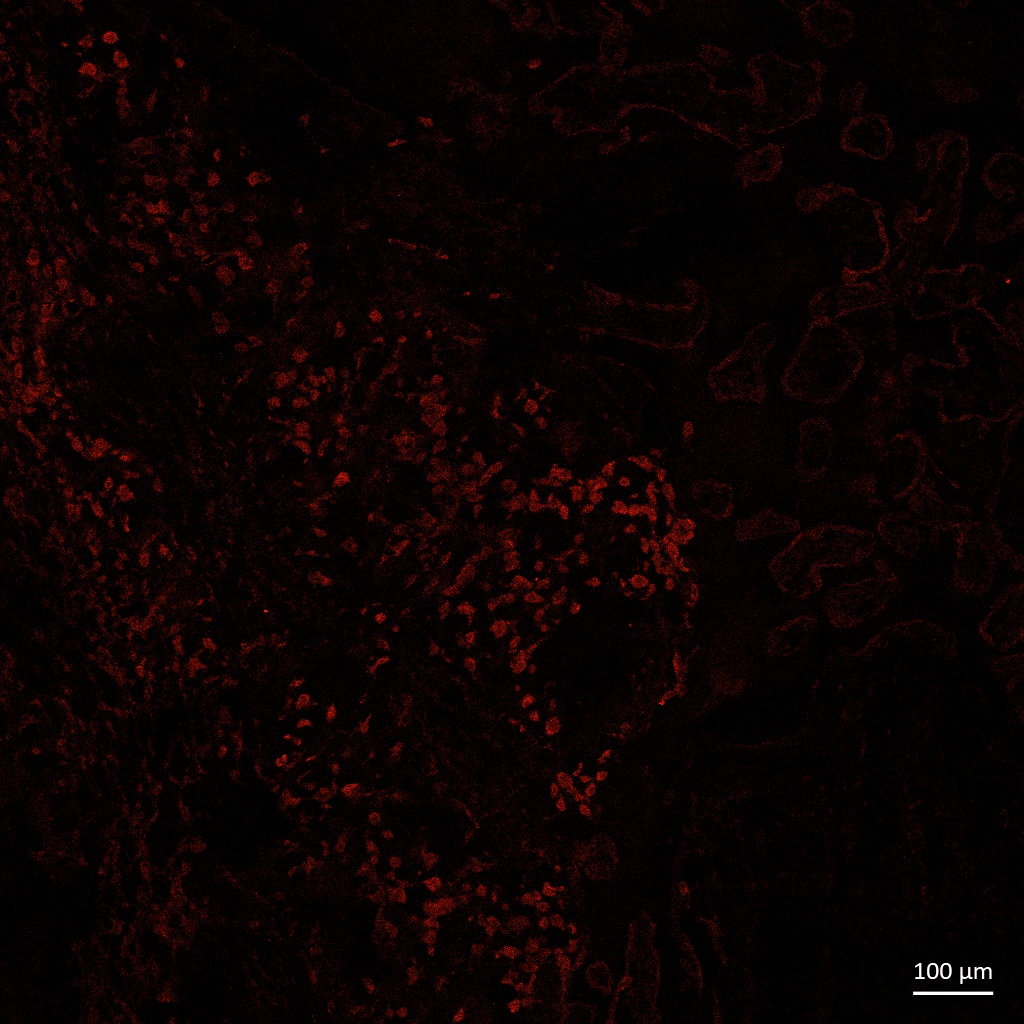

Supplement: Figure 2—source data 1. [file elife-100002-fig2-data1.zip › Figure 2-source data/figure 2D PE/PE-1.jpg]

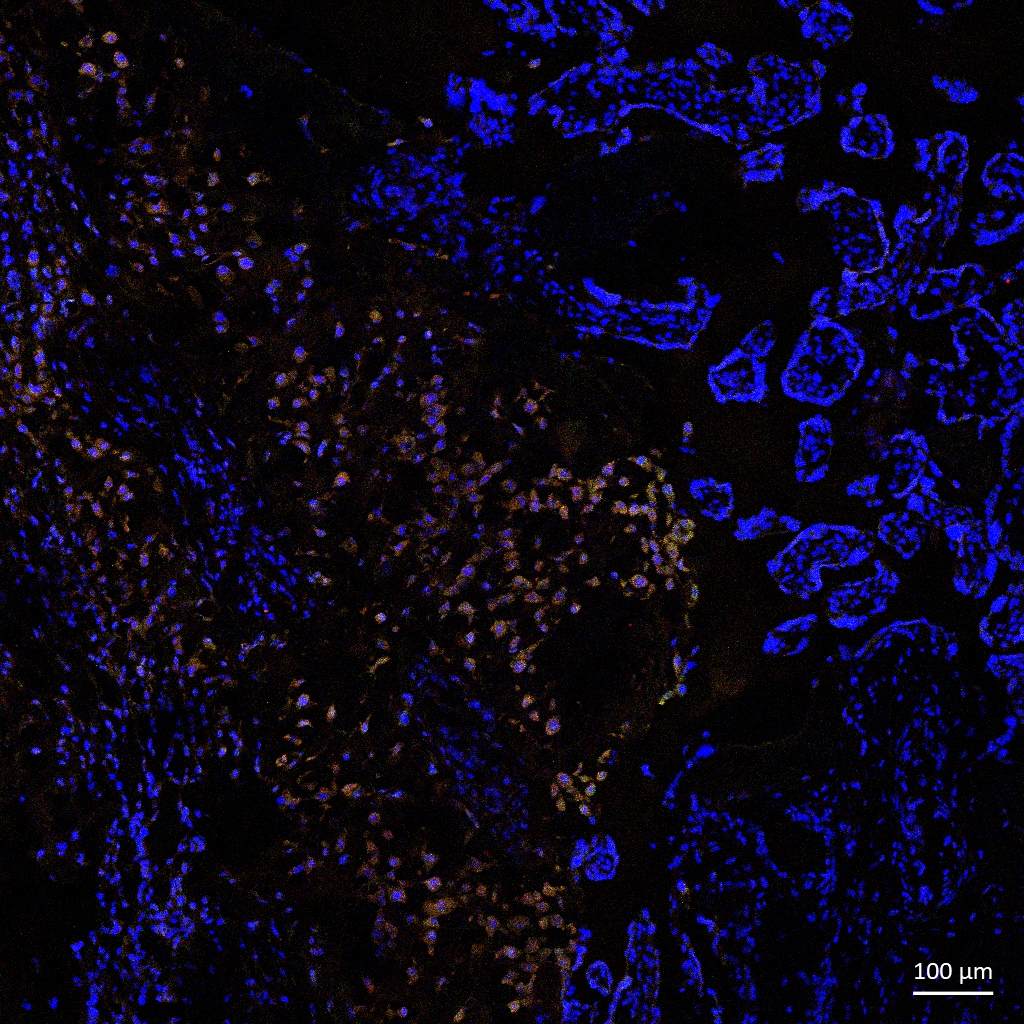

Supplement: Figure 2—source data 1. [file elife-100002-fig2-data1.zip › Figure 2-source data/figure 2D PE/PE-2.jpg]

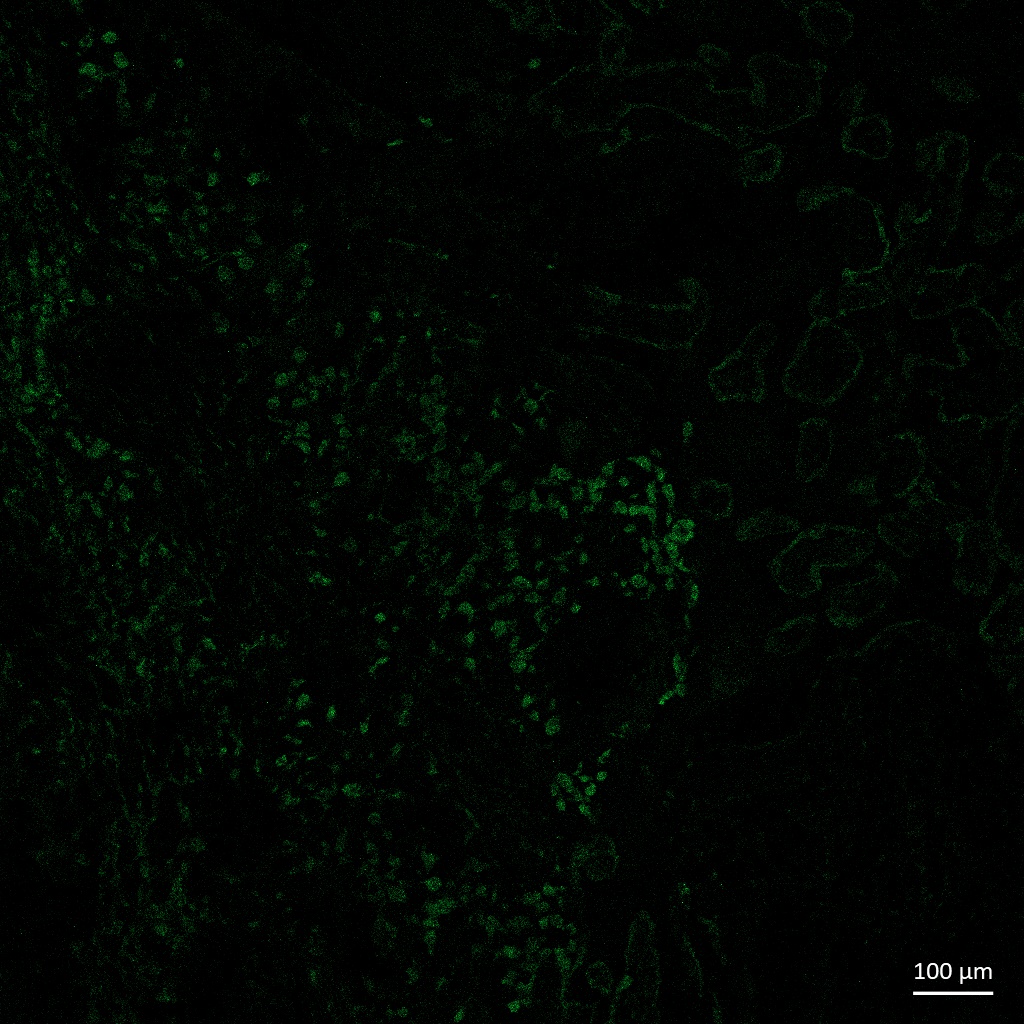

Supplement: Figure 2—source data 1. [file elife-100002-fig2-data1.zip › Figure 2-source data/figure 2D PE/PE-3.jpg]

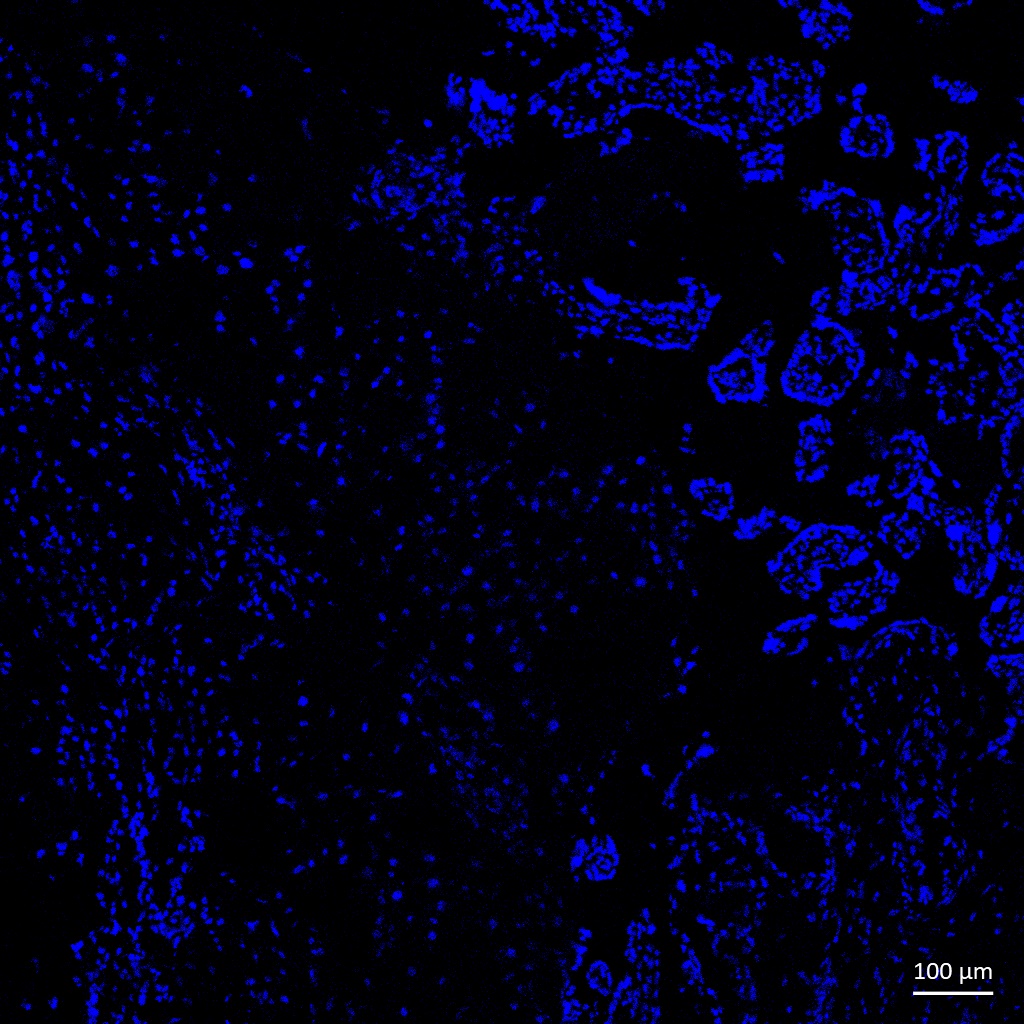

Supplement: Figure 2—source data 1. [file elife-100002-fig2-data1.zip › Figure 2-source data/figure 2D PE/PE-4.jpg]
